# Mid-route pancreatic duct seeking during endoscopic ultrasound-guided pancreatic duct drainage

**DOI:** 10.1055/a-2951-0080

**Published:** 2026-09-11

**Authors:** Soma Fukuda, Masato Endo, Yusuke Niisato, Yuya Hagiwara, Takashi Ishikawa, Taku Sakamoto, Kiichiro Tsuchiya

**Affiliations:** 1 Department of Gastroenterology, Institute of Medicine 38515 University of TsukubaTsukubaIbarakiJapan; 2 Graduate School of Comprehensive Human Sciences 13121 University of TsukubaTsukubaIbarakiJapan

Endoscopic ultrasound–guided pancreatic duct drainage (EUS-PDD) for a nondilated pancreatic duct is challenging because it requires a fine needle and fine-caliber guidewire, resulting in limited support for device insertion. 1​ A steep puncture angle can further impede device advancement. 2​ Although guidewire exchange or the double-guidewire technique may improve device insertability, 3​ ​4​ both require initial catheter insertion. We report a case in which guidewire manipulation from the mid-portion of the endosonographically created route (ESCR), 5​ which we termed mid-ESCR pancreatic duct seeking, facilitated pancreatic duct access after failed device insertion during EUS-PDD ( Video 1 ).

**Video 1** Mid-route pancreatic duct seeking using an uneven double-lumen cannula during endoscopic ultrasound-guided pancreatic duct drainage.


A 60-year-old man developed a pancreatic fistula due to pancreaticojejunal anastomotic leakage after pancreaticoduodenectomy for bile duct cancer. Computed tomography showed a fluid collection around the anastomosis, and percutaneous drainage was performed. Contrast injection confirmed communication between the pancreatic fistula and the pancreatic duct, without communication with the afferent limb ( Fig. 1 ). Because retrograde stenting via the afferent limb was considered difficult, EUS-PDD was attempted.

**Fig. 1:**
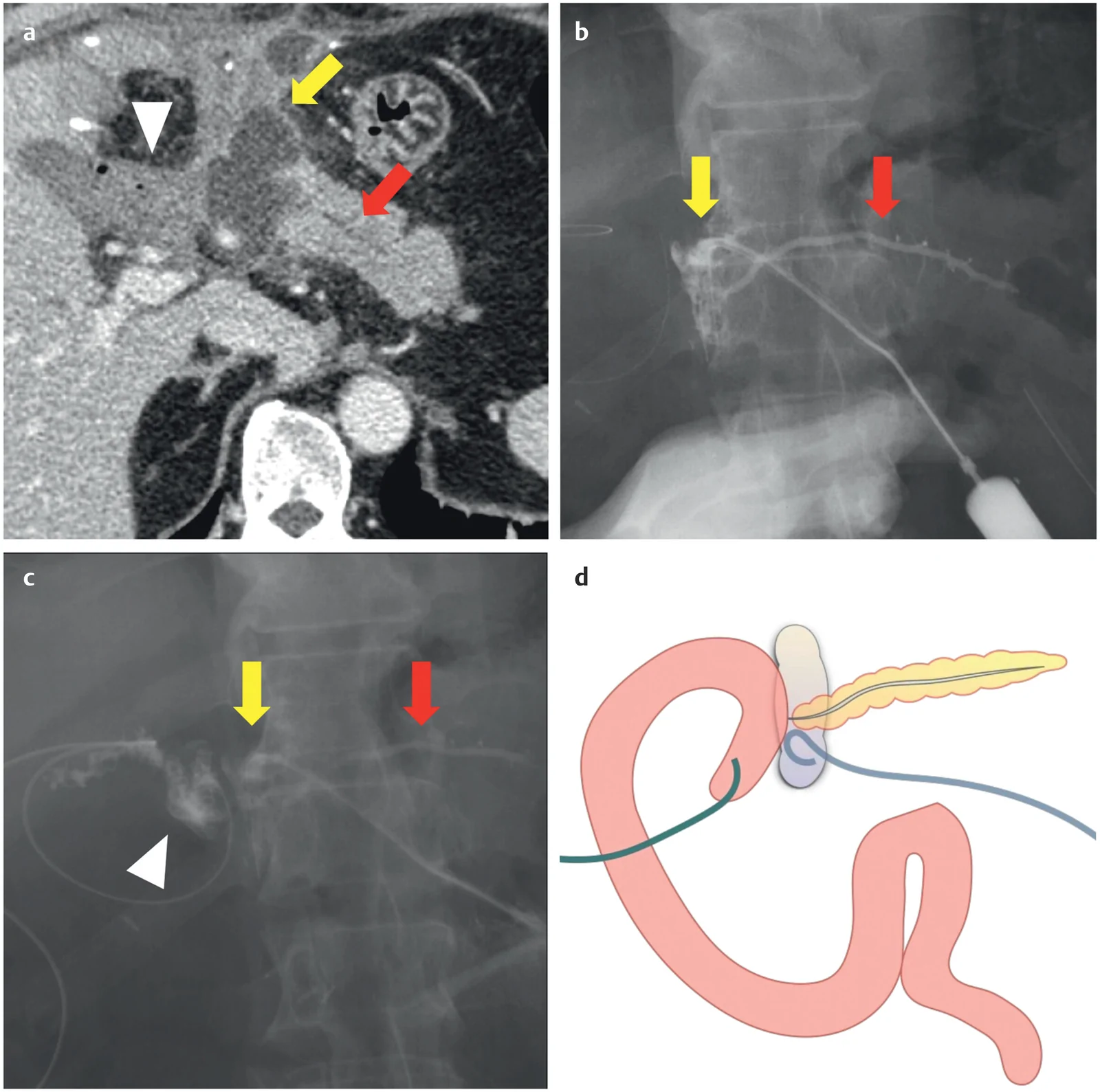
Preprocedural findings. ( **a** – **c** ) White arrowhead, afferent limb; yellow arrow, fluid collection; red arrow, main pancreatic duct. ( **a** ) Computed tomography showed a fluid collection around the pancreaticojejunal anastomosis. ( **b** and **c** ) Percutaneous drainage catheter contrast opacified the main pancreatic duct, whereas afferent limb catheter contrast confirmed separation from the pancreatic duct. ( **d** ) Schematic of the pancreatic fistula due to pancreaticojejunal anastomotic leakage.

A 1.5-mm main pancreatic duct was punctured with a 22-gauge needle, and a 0.018-inch guidewire was placed ( Fig. 2a, b ). The ESCR was dilated using a drill dilator ( Fig. 2c ). However, the 0.018-inch guidewire-compatible uneven double-lumen cannula (UDLC) could not be advanced because it failed to cross the pancreatic duct wall, causing tract buckling and cavity formation ( Fig. 3a, b ). The UDLC was therefore positioned in the mid-portion of the ESCR, above the pancreatic duct wall ( Fig. 3c ). Under fluoroscopic guidance, a 0.025-inch guidewire was advanced through the side lumen into the pancreatic duct, creating a double-guidewire configuration ( Fig. 3d, e ). This provided sufficient support for UDLC advancement ( Fig. 3f ). The guidewire was passed across the pancreaticojejunal anastomosis, and a 6-Fr, 15-cm pancreatic stent was deployed ( Fig. 4 ).

**Fig. 2:**
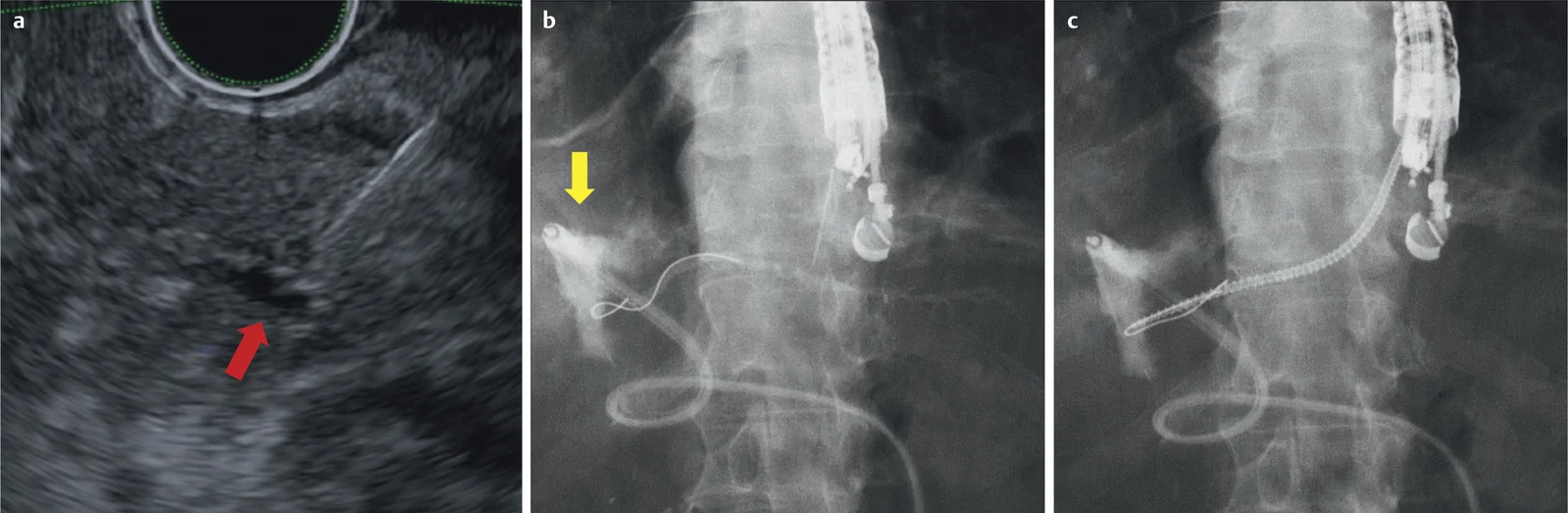
Initial endoscopic ultrasound (EUS)–guided pancreatic duct access. ( **a** ) An EUS image showing the 1.5-mm main pancreatic duct (red arrow). ( **b** ) The pancreatic duct was punctured with a 22-gauge needle, and a 0.018-inch guidewire was advanced into the pancreatic duct. The yellow arrow indicates the pancreatic fistula. ( **c** ) The endosonographically created route (ESCR) was dilated using a 7-Fr drill dilator.

**Fig. 3:**
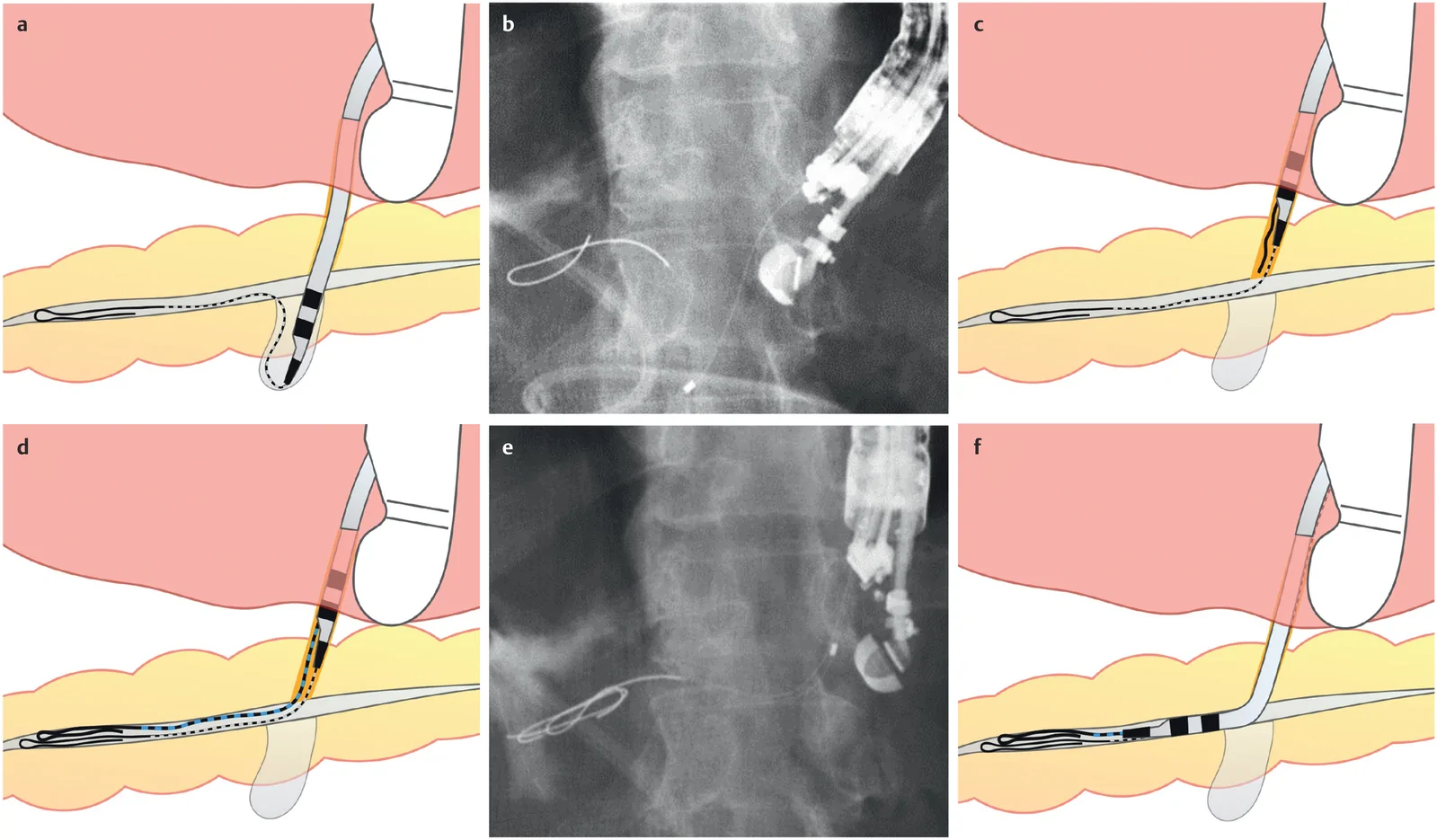
Mid-ESCR pancreatic duct seeking. ( **a** and **b** ) Failure to cross the pancreatic duct wall caused buckling of the endosonographically created route (ESCR) and cavity formation, preventing catheter advancement. ( **c** – **e** ) A 6-Fr uneven double-lumen cannula was positioned in the mid-portion of the ESCR, and a 0.025-inch guidewire was advanced through the side lumen into the pancreatic duct. ( **f** ) The double-guidewire configuration enabled advancement of the 0.025-inch guidewire-compatible cannula.

**Fig. 4:**
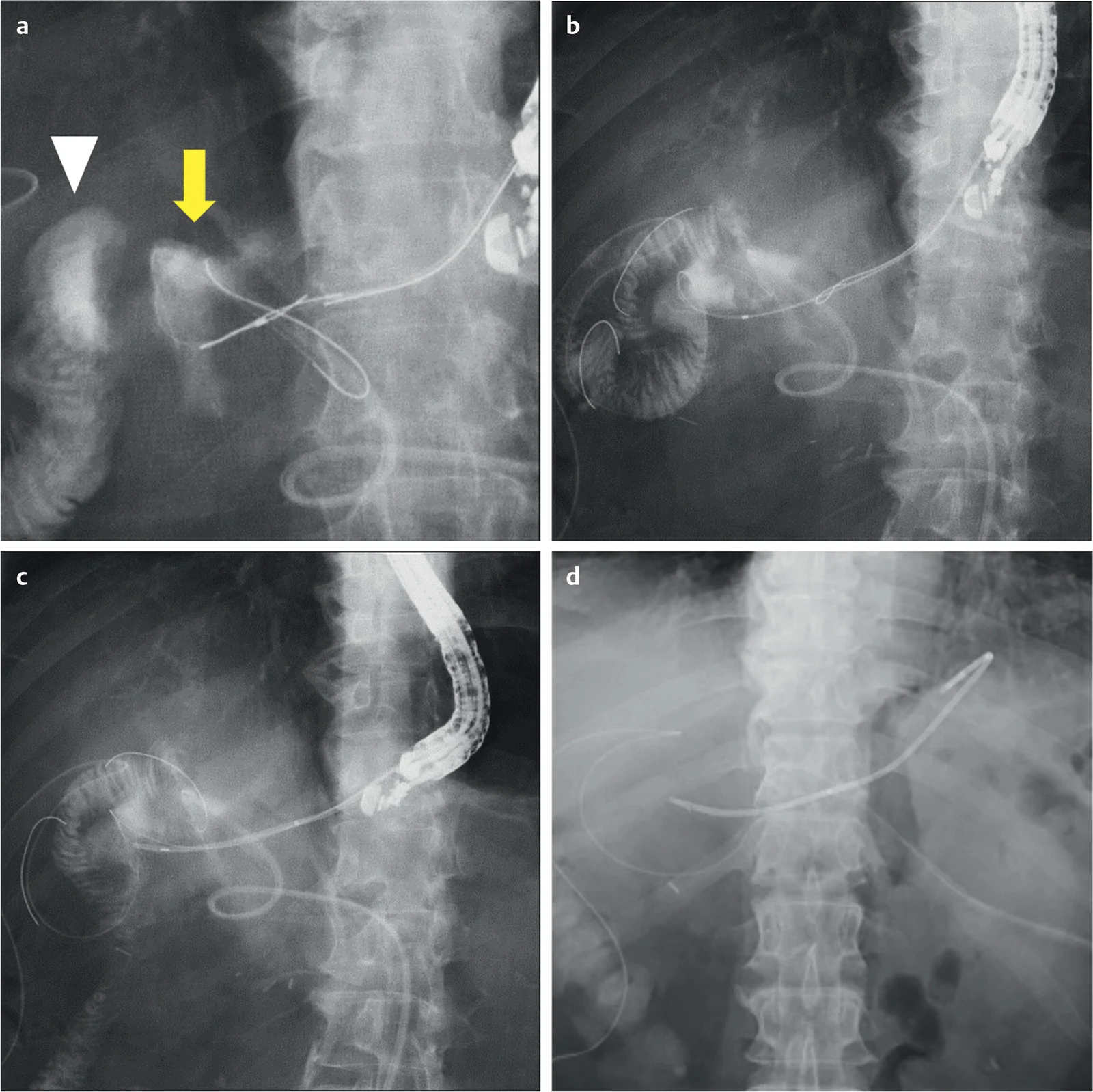
Successful device insertion and pancreatic stent deployment. ( **a** ) The double-guidewire configuration enabled advancement of the 0.025-inch guidewire-compatible uneven double-lumen cannula. The white arrowhead and yellow arrow indicate the afferent limb and pancreatic fistula, respectively. ( **b** ) The guidewire was passed across the pancreaticojejunal anastomosis. ( **c** ) A 6-Fr, 15-cm pancreatic stent was advanced across the anastomosis. ( **d** ) The final fluoroscopic image after stent deployment.

Computed tomography showed a small fluid collection around the stent, but the patient remained asymptomatic ( Fig. 5a, b ). The fluid collection resolved 1 month later ( Fig. 5c ). Mid-ESCR pancreatic duct seeking is an effective troubleshooting technique when the device insertion fails during EUS-PDD.

**Fig. 5:**
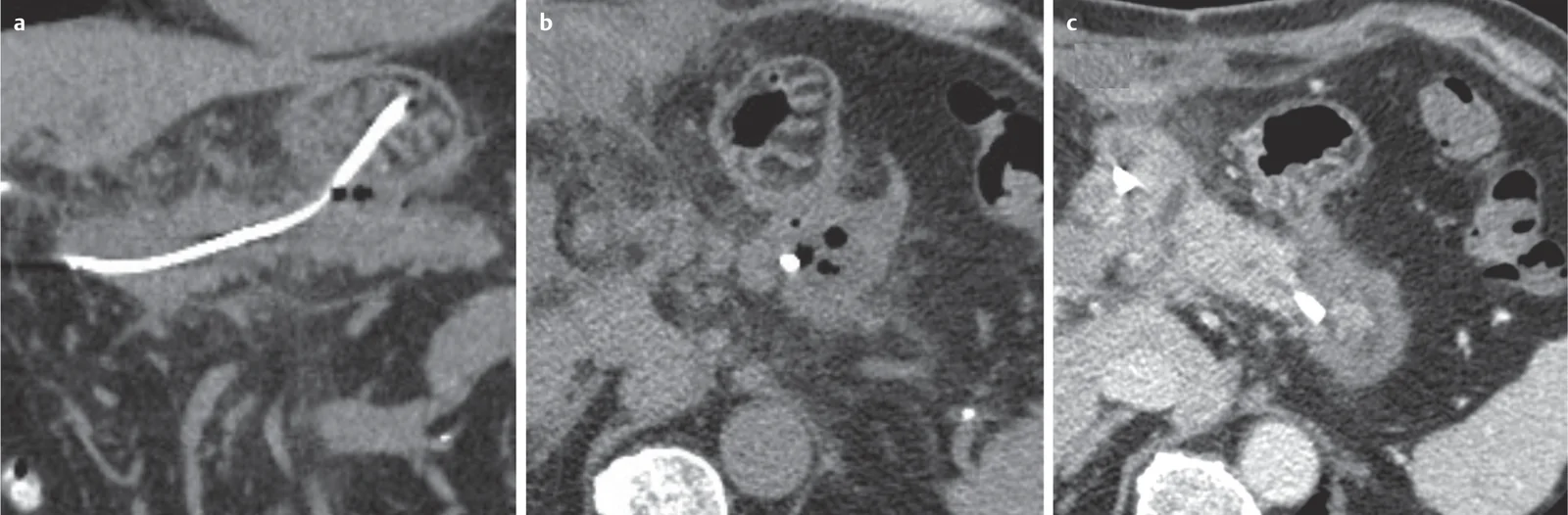
Postprocedural computed tomography findings. ( **a** ) The pancreatic stent was placed across the pancreaticojejunal anastomosis. ( **b** ) A small fluid collection with minimal air bubbles was seen around the stent. ( **c** ) The fluid collection resolved spontaneously 1 month later.

## Declaration of generative AI Use

The authors confirm that no Generative AI was used in the preparation of this manuscript.

Endoscopy_UCTN_Code_TTT_1AS_2AK
